# scRNA-seq profiling of neonatal and adult thymus-derived CD4^+^ T cells by a T cell origin-time tracing model

**DOI:** 10.1093/jmcb/mjac072

**Published:** 2022-12-20

**Authors:** Yuheng Han, Xinxing Ouyang, Yao Chen, Shujing Lai, Hongxiang Sun, Ningbo Wu, Chun Ruan, Limin Lu, Bing Su

**Affiliations:** Shanghai Institute of Immunology, Department of Immunology and Microbiology, and the Ministry of Education Key Laboratory of Cell Death and Differentiation, Shanghai Jiao Tong University School of Medicine, Shanghai 200025, China; Shanghai Institute of Immunology, Department of Immunology and Microbiology, and the Ministry of Education Key Laboratory of Cell Death and Differentiation, Shanghai Jiao Tong University School of Medicine, Shanghai 200025, China; Department of Tumor Biology, Shanghai Chest Hospital, Shanghai Jiao Tong University School of Medicine, Shanghai 200030, China; Shanghai Institute of Immunology, Department of Immunology and Microbiology, and the Ministry of Education Key Laboratory of Cell Death and Differentiation, Shanghai Jiao Tong University School of Medicine, Shanghai 200025, China; Shanghai Institute of Immunology, Department of Immunology and Microbiology, and the Ministry of Education Key Laboratory of Cell Death and Differentiation, Shanghai Jiao Tong University School of Medicine, Shanghai 200025, China; Shanghai Institute of Immunology, Department of Immunology and Microbiology, and the Ministry of Education Key Laboratory of Cell Death and Differentiation, Shanghai Jiao Tong University School of Medicine, Shanghai 200025, China; Shanghai Institute of Immunology, Department of Immunology and Microbiology, and the Ministry of Education Key Laboratory of Cell Death and Differentiation, Shanghai Jiao Tong University School of Medicine, Shanghai 200025, China; Shanghai Institute of Immunology, Department of Immunology and Microbiology, and the Ministry of Education Key Laboratory of Cell Death and Differentiation, Shanghai Jiao Tong University School of Medicine, Shanghai 200025, China; Shanghai Institute of Immunology, Department of Immunology and Microbiology, and the Ministry of Education Key Laboratory of Cell Death and Differentiation, Shanghai Jiao Tong University School of Medicine, Shanghai 200025, China; Shanghai Institute of Immunology, Department of Immunology and Microbiology, and the Ministry of Education Key Laboratory of Cell Death and Differentiation, Shanghai Jiao Tong University School of Medicine, Shanghai 200025, China; Center for Human Translational Immunology at Shanghai Institute of Immunology, Ruijin Hospital, Shanghai Jiao Tong University School of Medicine, Shanghai 200025, China; Shanghai Jiao Tong University School of Medicine–Yale Institute for Immune Metabolism, Shanghai Jiao Tong University School of Medicine, Shanghai 200025, China; Key Laboratory of Molecular Radiation Oncology of Hunan Province, Xiangya Hospital, Central South University, Changsha 410008, China

**Keywords:** T lineage tracing, Treg, neonatal thymus-derived T cells, neonatal thymus-derived Treg, single-cell RNA sequencing

## Abstract

It is well documented that the neonatal thymus-derived (neonatal-TD) regulatory T cells (Treg) are essential to prevent lethal autoimmune diseases and allergies, and neonatal and adult thymus possesses distinct output potentials for naïve T cells, including Treg. However, the molecular features and detailed functional differences between neonatal-TD and adult thymus-derived (adult-TD) T cells in terms of their ability to maintain immune homeostasis during long-term environmental influences are still largely unknown, partially due to the lack of appropriate animal models to precisely trace these cells at specific time points. In this study, neonatal-TD and adult-TD CD4^+^ T cells from the spleen and Peyer's patches were traced for 9 weeks by a T cell origin-time tracing mouse model and analysed by single-cell RNA sequencing. More Treg but fewer naïve T cells were found in neonatal-TD CD4^+^ T cells from both tissues than those from adult-TD counterparts. Interestingly, the neonatal-TD Treg in both the spleen and Peyer's patches exhibited augmented expression of *Foxp3, Gata3, Ctla4, Icos, Il2ra, Tgfb1*, and *Nrp1*, as well as enriched Gene Ontology terms like T cell activation and tolerance induction, indicating an enhanced immunosuppressive function. These results were further confirmed by flow cytometry analysis and *in vitro* immune suppression assays. Flow cytometry also revealed a significantly higher proportion of neonatal-TD Treg in total Treg than that of adult-TD counterparts, suggesting the longer lifespan of neonatal-TD Treg. To investigate the intrinsic features of neonatal-TD and adult-TD CD4^+^ T cells, a shortened tracing time was performed. Surprisingly, the neonatal-TD and adult-TD CD4^+^ T cells had similar proportions of Treg and did not exhibit significant differences in *Foxp3, Gata3, Ctla4, Icos, Il2ra*, and *Tgfb1* expression levels after tracing for 12 days. On the other hand, neonatal-TD Treg present an increased *Nrp1* expression level compared with adult-TD counterparts, indicating the enhanced stability. Together, our work reveals that the neonatal-TD Treg are more immunosuppressive, which is likely shaped primarily by environmental factors.

## Introduction

Neonatal T cells are known to exhibit weak cellular immune responses but stronger immunosuppressive properties (Adkins et al., 2004). In the 1980s and 1990s, researchers revealed that neonatal CD4^+^ T cells preferentially produced cytokines of T helper 2 cells (Th2), but made fewer cytokines of T helper 1 cells (Th1) after stimulation (Forsthuber et al., 1996). These findings indicated that neonatal T cells were not immature or dysfunctional; instead, they had different immune responses compared with their adult counterparts. Furthermore, CD4^+^ T cells of neonatal mice exhibit rapid proliferation and a bias to differentiate into Th2 and regulatory T cells (Treg), but not Th1, T helper type 17 cells (Th17), or T follicular helper cells (Tfh) after stimulation (Wang et al., 2010; Debock and Flamand, 2014).

During the neonatal period, establishing tolerance to self-antigens, food antigens, and microbe antigens is essential to avoid autoimmunity and allergies, and Treg play an important role in this process (Yang et al., 2020). Treg prevent autoimmune diseases and tissue damage caused by misdirected and excessive immune reactions in response to self and foreign targets by secreting immunosuppressive cytokines and engaging in cell surface interactions (Josefowicz et al., 2012). Treg deficiency leads to severe autoimmune diseases and inflammatory diseases; Treg dysfunction, especially during the fetal and neonatal periods, results in the onset of IPEX syndrome in the second-trimester in Foxp3-deficient humans (Chatila, 2005; Bacchetta et al., 2018). Neonatal Treg have an enhanced proliferative ability in both humans and mice (Yang et al., 2015; Hayakawa et al., 2017). Treg depletion in neonatal mice by the Foxp3^DTR^ model quickly results in severe autoimmune phenotypes, but when Treg are depleted during the adult period, only mild autoimmune phenotypes are evident (Yang et al., 2015). Unlike continuous Treg depletion by diphtheria toxin during the neonatal period, two injections of low-dose diphtheria toxin in neonatal Foxp3^DTR^ mice do not cause severe autoimmunity but lead to dysregulation of subcutaneous tissue in the skin and selective accumulation of Th2 in a unique microanatomical niche (Boothby et al., 2021). Neonatal Treg can be recruited to tissues to suppress immunoreactions (Yang et al., 2020). Treg from the neonatal thymus enter the liver, and depletion of these Treg between Days 7 and 11 leads to Th1-type liver inflammation (Li et al., 2020). A wave of unique neonatal Treg can be recruited to neonatal skin and hair follicles in 1- to 2-week-old mice colonized with the *Staphylococcus epidermidis*, and tolerance to the microbe can be established during the neonatal period, but not the adult period. Selective inhibition of the neonatal Treg wave leads to abrogation tolerance (Scharschmidt et al., 2015, 2017). These studies demonstrated that neonatal Treg are crucial for the establishment and maintenance of immunotolerance.

There are many reports about the uniqueness of neonatal CD4^+^ T cells, but the characteristics of T cells originating from neonates and their persisting roles in adulthood compared to their adult counterparts over the same time are still unclear. This is because no suitable tools exist to separate these two groups of cells for analysis. Here in this study, a T cell origin-time tracing (TOTT) mouse model was specifically used to mark the birth time of neonatal thymus-derived (neonatal-TD) and adult thymus-derived (adult-TD) T cells in the periphery and allow tracing them *in vivo* for different periods (Zhang et al., 2015). This model has been used to reveal the existence of long-live T cell clones and the enrichment of shared clonotypes between the non-Treg and Treg clones, indicating the involvement of common antigens in selecting the retention of long-live T cell clones (Zhang et al., 2016).

Using the TOTT model, we analysed the T cell subsets and their respective gene expression patterns of neonatal-TD and adult-TD CD4^+^ T cells in the spleen and Peyer's patches after tracing for 9 weeks by single-cell RNA sequencing (scRNA-seq). We found that Treg of neonatal-TD had higher expression levels of *Foxp3* and other Treg signature genes (*Gata3, Ctla4, Icos, Il2ra, Tgfb1*, and *Nrp1*), as well as enriched Gene Ontology (GO) terms (T cell activation and tolerance induction), indicating an enhanced immunosuppressive function. The increased expression levels of genes (*Icos, Tnfrsf9*, and *Tnfrsf18*) associated with enhanced proliferation and survival were also found in neonatal-TD Treg compared with adult-TD Treg. Neonatal-TD T cells in the Peyer's patches had Treg features and enriched GO terms that were similar to neonatal-TD Treg in the spleen, compared with adult-TD Treg. Flow cytometry analysis revealed a significantly higher proportion of neonatal-TD Treg in total Treg than that of adult-TD counterparts, indicating the increased longevity of neonatal-TD Treg. Flow cytometry analysis also confirmed an increased proportion of Treg in ZsGreen^+^CD4^+^ T cells and higher expression levels of Foxp3 and CD25, and the co-culture experiment demonstrated the enhanced immunosuppressive ability of neonatal-TD Treg.

To determine the environmental influence on the fate of neonatal-TD and adult-TD T cells, a short 12-day-tracing experiment was also performed. The short-term-traced neonatal-TD T cells contained a similar proportion of Treg to that in short-term-traced adult-TD T cells. Unlike Treg after long-term tracing, short-term-traced neonatal-TD and adult-TD Treg did not exhibit significant differences in the expression levels of *Foxp3, Gata3, Ctla4, Icos, Il2ra, Tgfb1, Tnfrsf9*, and *Tnfrsf18*, but *Nrp1* expression level was higher in neonatal-TD Treg than in adult-TD Treg, which indicated that neonatal-TD Treg are more stable than adult-TD Treg. Our results indicate that the increased immunosuppressive ability of 9-week-traced neonatal-TD Treg is likely shaped primarily by environmental factors.

## Results

### T Cells originated at a specific time point from the thymus can be effectively traced by the TOTT model

**Figure 1:**
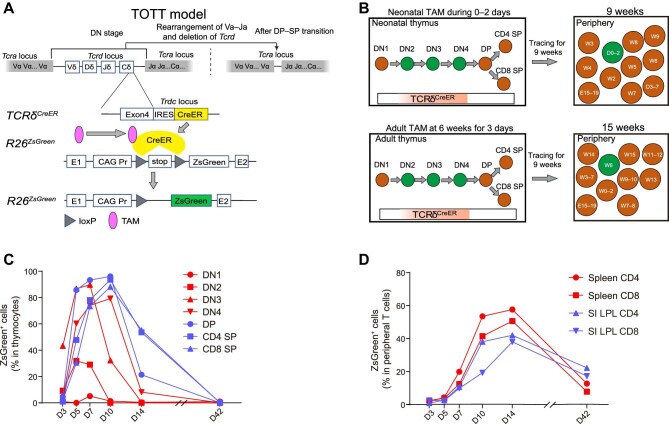
Tracing and flow cytometry analysis of neonatal-TD CD4^+^ T cells by the TOTT mouse model demonstrate the efficacy of the model. (**A**) Schematic presentation of the TOTT model and its response to tamoxifen treatment. The *Tcrd* gene transcribes during the DN2–DN4 stages in T cell development. After the V–J rearrangement during the DP–SP transition, the *Tcrd* locus is deleted and the CreER fusion protein never transcribes. CreER is inactive in DN2–DN4 thymocytes without tamoxifen. During tamoxifen treatment, CreER in DN2–DN4 thymocytes is activated to excise the loxP–stop–loxP cassette and allow the expression of ZsGreen. Pr, promoter; TAM, tamoxifen; *Trdc*, T cell receptor delta constant. (**B**) Workflow of the TOTT mouse model during and after tamoxifen treatment. For tamoxifen treatment during neonatal 0–2 days, DN2–DN4 thymocytes were labelled with ZsGreen to mark their birth time. After tracing for 9 weeks, the birth time of ZsGreen^+^ cells was restricted to the period of neonatal 0–2 days, while that of ZsGreen-negative cells covered the whole life course. For tamoxifen treatment at 6 weeks, DN2–DN4 thymocytes were labelled with ZsGreen at 6 weeks. After tracing for 9 weeks, the birth time of ZsGreen^+^ cells was restricted to 6 weeks, while that of ZsGreen-negative cells was from the prenatal to the adult period. E15–19, embryonic day 15–19. (**C** and **D**) Mice were treated with tamoxifen during neonatal 0–2 days, and each sample was a mixture from three mice. (**C**) Proportion of ZsGreen^+^ neonatal-TD cells in each subset of thymocytes. (**D**) Proportion of ZsGreen^+^ neonatal-TD cells in CD4^+^ and CD8^+^ T cells of the spleen and SI LPL.

The current Cre transgenic strains with thymus-initiated promoters (such as *Lck* and *Cd4*) not only target developing thymic T cells but also affect peripheral mature T cells. These tools cannot divide T cells based on their origin at a specific time point. To investigate the signatures of neonatal-TD Treg, we needed to distinguish neonatal-TD T cells from other T cells. With the TOTT model, which has been described previously, we were able to trace the T cell origin from a specific time point (Zhang et al., 2015). Briefly, the TOTT model is constituted by crossing the *TCRδ^CreER^* strain with the *R26^ZsGreen^* reporter strain (Figure 1A). The *Tcrd* gene is expressed in αβT cells beginning from CD4^−^CD8^−^ double-negative-2 (DN2) stage, and is deleted upon the completion of *TCR*α gene rearrangement during the transition from the CD4^+^CD8^+^ double-positive (DP) stage to the CD4^+^ or CD8^+^ single-positive (SP) stage, suggesting that the model never labels mature αβT cells (Prinz et al., 2006; Zhang et al., 2016). After tamoxifen treatment, the CreER protein expressed in the DN2–DN4 thymocytes is transiently activated to delete the loxP–stop–loxP cassette in *R26^ZsGreen^* reporter and allow the expression of ZsGreen to mark the age of T cells at this stage (Figure 1A and B). Consequently, the T cells that originate during tamoxifen treatment are marked by the ZsGreen label, while the other T cells are nonfluorescent. This means that the birth time of ZsGreen-labelled cells was restricted to the period of tamoxifen treatment, while that of ZsGreen-negative cells was from the prenatal to the adult period (Figure 1B). By the model, we can directly trace and compare T cells produced from different time points during the life course.

To test the efficiency of the TOTT model, neonatal mice were treated with tamoxifen during neonatal 0–2 days. The suspensions of thymocytes, splenocytes, and small intestine (SI) lamina propria lymphocytes (LPL) were isolated and stained with an antibody cocktail containing anti-TCRβ, anti-TCRγδ, anti-CD4, and anti-CD8 (anti-CD44 and anti-CD25 were additionally used in the cocktail for thymocytes). The proportions of ZsGreen^+^ cells in thymus DN1–DN4, DP, CD4^+^/CD8^+^ SP cells, and CD4^+^/CD8^+^ T cells in the spleen and LPL (all thymocytes and CD8^+^ T cells were gated from TCRγδ^–^ cells, DN1 cells were TCRγδ^–^TCRβ^–^CD4^–^CD8^–^CD44^+^CD25^–^) were examined on Days 3, 5, 7, 10, 14, and 42 by flow cytometry analysis. In the thymus, DN1 cells were not marked by ZsGreen as expected, and ZsGreen^+^ DN2 cells peaked around Day 5, which suggested that tamoxifen treatment marked T cells during Days 3–5 (Figure 1C). ZsGreen^+^ DN3 cells peaked around Day 7, and ZsGreen^+^ DN4, DP, and CD4^+^/CD8^+^ SP cells peaked on Day 10. DP cells contained up to 96% ZsGreen^+^ cells, which confirmed the high efficiency of the TOTT model (Figure 1C). In the spleen and LPL, ZsGreen^+^TCRβ^+^CD4^+^/CD8^+^ T cells reached the highest percentage around Day 14 and still existed on Day 42 (Figure 1D). The results confirm that we can trace T cells originated from the thymus at a specific time point by the TOTT model, and these neonatal labelled cells can survive in the periphery for a long time as reported in the previous study (Zhang et al., 2016).

### Neonatal-TD splenic T cells enrich more Treg, Tm, and Th17 but fewer Tn

To investigate the subset heterogeneity and functional differences between neonatal-TD and adult-TD CD4^+^ T cells, neonatal TOTT mice were treated with tamoxifen during 0–2 days after birth whereas adult TOTT mice were treated at 6 weeks old for 3 days. Neonatal-TD and adult-TD CD4^+^ T cells from the spleen, which were ZsGreen^+^, were sorted 9 weeks later (Figure 2A; Supplementary Figure S1A and B). The purity of the sorted CD4^+^ZsGreen^+^ cells was checked by flow cytometry after fluorescence-activated cell sorting (FACS), and the neonatal-TD 9-week-traced spleen (neonatal-TD 9W SPL, 4004 cells) and adult-TD 9-week-traced spleen (adult-TD 9W SPL, 16317 cells) were immediately processed for scRNA-seq (Supplementary Figure S1C). After filtering out the low-quality cells, cell doublets, and non-CD4^+^ T cells from the data, the transcriptomes of a total of 14203 cells from the neonatal-TD 9W SPL (3561 cells) and adult-TD 9W SPL (10642 cells) were obtained for subsequent analysis. After gene expression normalization for sequencing depth and mitochondrial read count, the principal component analysis (PCA) was then applied for primary dimension reduction. The scRNA-seq data were integrated with the Harmony package to correct the batch effect. We further generated a unified Uniform Manifold Approximation and Projection (UMAP) embedding space with the Harmony-corrected principal components and then performed graph-based clustering (Figure 2B). The purity was examined by the expression of the CD4^+^ T cell marker genes *Cd3g* and *Cd4*. As expected, this population of cells was all CD4^+^ T cells, without the expression of other subset signature genes including *Cd8a* for CD8^+^ T cells, *Ms4a1* for B cells, and *Fcgr4* and *Cd14* for monocytes (Supplementary Figure S2; Heng et al., 2008). CD4^+^ T cells from both neonatal-TD 9W SPL and adult-TD 9W SPL were segregated into seven distinct subsets, including naïve T cells (Tn), Treg, memory T cells (Tm), T helper type 1 cells (Th1), Th17, Tfh, and unknown clusters. Tn were identified by *Sell*, encoding a membrane protein CD62L, and *Lef1*, encoding transcription factor LEF1 (Heng et al., 2008). Treg were characterized by *Foxp3*, encoding transcription factor Foxp3, and *Ikzf2*, encoding transcription factor Helios (Heng et al., 2008). Tm were recognized with the high expression of *S100a4*, encoding S100a4, which is exclusively expressed in Tm (Weatherly et al., 2015). Th1 were characterized by *Tbx21*, encoding transcription factor T-bet, and *Ifng*, encoding cytokine IFN-γ (Zhu et al., 2010). Th17 were identified by the enriched expression of *Rorc*, encoding transcription factor Rorγt, and *Il17a*, encoding cytokine IL-17 (Kumar et al., 2021). Tfh were identified by the enriched expression of *Pdcd1*, encoding immune checkpoint receptor PD1, and *Cxcr5*, encoding chemokine receptor CXCR5 (Vinuesa et al., 2016). UMAP highlighted a shift between Tn of neonatal-TD and adult-TD groups, which suggested the distinct gene expression patterns (Figure 2B). Specifically, we found that neonatal-TD 9W SPL cells showed an increase in proportions of Treg (14.32% vs. 9.48%), Tm (20.95% vs. 11.42%), Th17 (8.40% vs. 2.57%) and a decrease in the proportion of Tn (39.40% vs. 62.07%; Figure 2C). The signature scores were higher in neonatal-TD Treg and neonatal-TD Tn but lower in neonatal-TD Th1 than that in adult-TD counterparts in the spleen, revealing distinct gene expression patterns among neonatal and adult groups (Figure 2C). Then, we focused on Treg, Tn, Th1, and Th17 in the following study.

**Figure:**
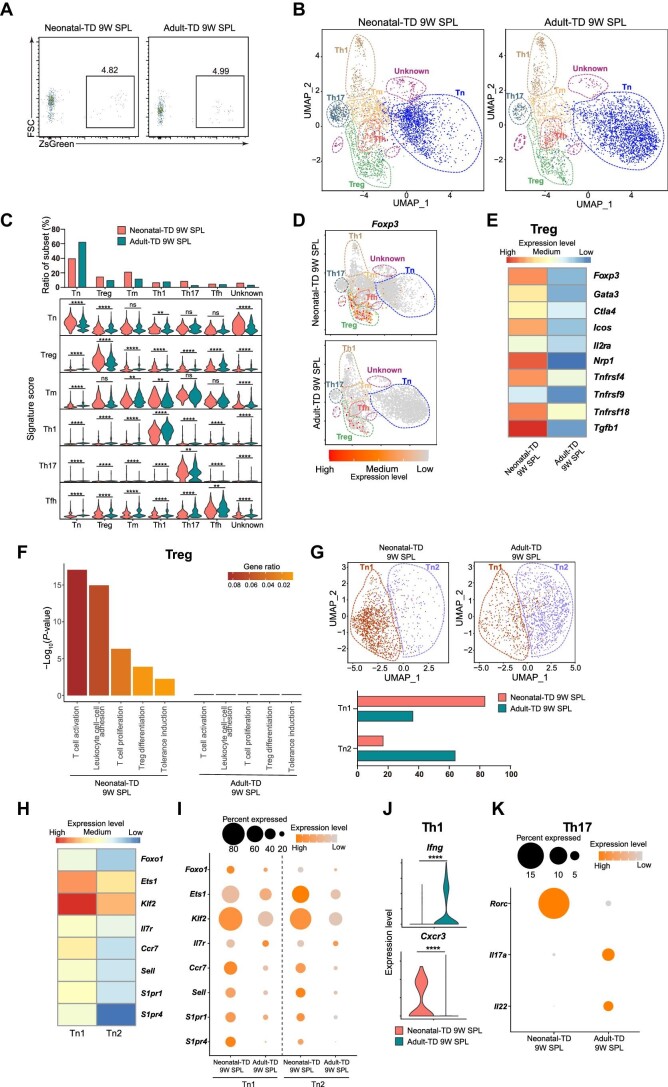
scRNA-seq reveals the differences in subset distribution and gene expression patterns between neonatal-TD and adult-TD CD4^+^ T cells in the spleen after tracing for 9 weeks. (**A**) Flow cytometry analysis of 9-week-traced neonatal-TD and adult-TD ZsGreen^+^CD4^+^ T cells in the spleen. (**B**) UMAP display of scRNA-seq data from CD4^+^ T cells of 9-week-traced neonatal-TD (3561 cells) and adult-TD (10642 cells) in the spleen. Cells were segregated into seven clusters, including Tn, Treg, Tm, Th1, Th17, Tfh, and unknown clusters. The cell number of each sample was equalized. (**C**) The top bar graph indicates the frequency of cells in each cluster from the neonatal-TD and adult-TD groups, and the bottom stacked violin plot displays signature scores of each subset from different groups. (**D**) Feature plots display *Foxp3* expression in T cells from the neonatal-TD and adult-TD groups. (**E**) Heatmap reveals distinct expression levels of the signature genes (*Foxp3, Gata3, Ctla4, Icos, Il2ra, Nrp1, Tnfrsf4, Tnfrsf9, Tnfrsf18*, and *Tgfb1*) in Treg of neonatal-TD 9W SPL and adult-TD 9W SPL. (**F**) Selected GO terms in neonatal-TD 9W SPL Treg compared with adult-TD 9W SPL Treg. Bars represent the GO terms using a discrete color scale with gene ratio. Bar length represents −log_10_(*P*-value) of the GO terms. (**G**) Top: UMAP visualization of scRNA-seq data from Tn of 9-week-traced neonatal-TD and adult-TD groups in **B**. The cell number of each sample was equalized. Bottom: the bar graph indicates the proportion of cells in each cluster from neonatal-TD Tn and adult-TD Tn. (**H**) Heatmap exhibits distinct expression levels of the selected genes (*Foxo1, Ets1, Klf2, Il7r, Ccr7, Sell, S1pr1*, and *S1pr4*) between Tn1 and Tn2. (**I**) Dot plot exhibits expression levels of the selected genes (*Foxo1, Ets1, Klf2, Il7r, Ccr7, Sell, S1pr1*, and *S1pr4*) in Tn1 and Tn2 from the neonatal-TD and adult-TD groups. (**J**) Violin plot presents expression levels of the selected genes (*Cxcr3* and *Ifng*) in Th1 of neonatal-TD 9W SPL and adult-TD 9W SPL. (**K**) Dot plot presents expression levels of the selected genes (*Rorc, Il17a*, and *Il22*) in Th17 of neonatal-TD 9W SPL and adult-TD 9W SPL.

### Neonatal-TD Treg express higher levels of immunosuppressive genes

Compared with their adult-TD counterparts, Treg of neonatal-TD 9W SPL had significantly increased expression levels of *Foxp3, Gata3, Ctla4, Icos, Il2ra, Nrp1, Tnfrsf4, Tnfrsf9, Tnfrsf18*, and *Tgfb1* (Figure 2D and E)*.* This gene expression pattern was associated with enhanced proliferation, survival, and immunosuppressive function. For example, Foxp3 is the key transcription factor that maintains the characteristic gene expression of Treg (Fontenot et al., 2003; Fontenot et al., 2005). Transcription factor Gata3 can promote the expression of Foxp3 by binding to the conserved noncoding sequence locus of *Foxp3*, and it can also enhance the immunosuppressive ability of Treg in Th2 and Th17 responses (Wang et al., 2011; Wohlfert et al., 2011). The *Il2ra* gene encodes CD25, which can suppress T cell activation and proliferation via competitive binding with IL-2 (Sakaguchi et al., 2020). In addition, CTLA4 can strongly suppress the T cell immune responses by binding to CD80/86 (Onishi et al., 2008; Chinen et al., 2016). Increased TGFβ expression can induce Tn to differentiate into Treg and suppress the function and numbers of Th1, Th2, and cytotoxic T lymphocytes (Travis and Sheppard, 2014). Increased *Icos* expression endows Treg with enhanced proliferation and survival abilities by enhancing the expression of Foxp3 (Chen et al., 2018). ICOS can also regulate Th2 responses by binding with ICOSL in Th2 (Shevyrev and Tereshchenko, 2019). OX40, 4-1BB, and GITR, which are encoded by *Tnfrsf4, Tnfrsf9*, and *Tnfrsf18*, respectively, can promote the proliferation and survival of Treg (Takeda et al., 2004; Zheng et al., 2004; van Olffen et al., 2009). Neuropilin-1 (Nrp1), encoded by *Nrp1*, can increase the immunosuppressive ability of Treg and stabilize Treg in tumors (Chuckran et al., 2020). Our results indicate the enhanced proliferation, survival, and immunosuppressive ability of neonatal-TD 9W SPL Treg.

To further compare the immunosuppressive ability of neonatal-TD 9W SPL Treg with adult-TD 9W SPL Treg, we performed the GO pathway analysis between the two groups. As expected, GO pathway terms, including T cell activation, leukocyte cell–cell adhesion, T cell proliferation, Treg differentiation, and tolerance induction, were enriched in neonatal-TD 9W SPL Treg, compared with their adult-TD counterparts (Figure 2F). T cell activation in Treg is associated with enhanced suppressive ability, and the cell–cell interaction of Treg with the effector T cells and antigen-presenting cells is essential to direct immune inhibition of Treg (Shevyrev and Tereshchenko, 2019; Sakaguchi et al., 2020). The enriched T cell proliferation indicated increased proliferation in neonatal-TD Treg, and the enriched Treg differentiation was associated with the increased Treg differentiation from Tn. These two terms were matched with the increased proportion of neonatal-TD Treg. Tolerance induction involved in neonatal-TD Treg suggested the enriched immunosuppressive gene expression. These data suggest the increased proportion and enhanced immunosuppressive ability of neonatal-TD Treg after tracing for 9 weeks.

Because of the distinct cell distributions between neonatal-TD 9W SPL Tn and adult-TD 9W SPL Tn, we separated Tn into two clusters (Tn1 and Tn2) and displayed the cells in the UMAP space (Figure 2G). Neonatal-TD Tn contained more Tn1 (83.32% vs. 36.16%) and less Tn2 (16.68% vs. 63.84%) than adult-TD Tn (Figure 2G). Tn1 were characterized by the enriched expression of *Foxo1, Ets1, Klf2, Il7r, Ccr7, Sell, S1pr1*, and *S1pr4*, and Tn2 had decreased expression of these genes (Figure 2H). *Foxo1* encodes transcription factor Foxo1, which controls the expression of Klf2, CD62L, and IL-7Rα, thereby regulating the homeostasis and life span of Tn (Kerdiles et al., 2009). Transcription factor Ets1, which is encoded by *Ets1*, maintains the expression of IL-7Rα in peripheral T cells (Grenningloh et al., 2011). IL-7Rα (encoded by *Il7r*) is critical for the long-term maintenance of T cells by modulating the pathway of apoptosis (Barata et al., 2019). Transcription factor Klf2 has an important role in the migration of Tn by controlling the expression of CD62L and S1pr1 (Cao et al., 2010). CD62L (encoded by *Sell*), S1pr1, and S1pr4 are essential for lymphocyte homing to lymphoid tissues and sites of inflammation (Cao et al., 2010; Xiong et al., 2019). Both Tn1 and Tn2 of neonatal-TD expressed higher levels of *Foxo1, Ets1, Klf2, Il7r, Ccr7, Sell, S1pr1*, and *S1pr4* than their adult-TD counterparts (Figure 2I). These results indicate that neonatal-TD 9W SPL Tn have an enhanced survival and migrating ability.

As for Th1, we found that adult-TD Th1 expressed more *Ifng*, but neonatal-TD Th1 expressed more *Cxcr3* (Figure 2J). Cxcr3 expressed on Th1 recruits these cells into inflamed tissues, and IFN-γ is the main cytokine involved in the Th1 responses (Groom and Luster, 2011). The different gene expression patterns between neonatal-TD and adult-TD Th1 indicate their distinct Th1 responses.

In Th17, we found that adult-TD Th17 had higher expression levels of *Il17a* and *Il22* but lower expression level of *Rorc* (Figure 2K). IL-17 protects the barrier surface tissues from environmental threats through inducing epithelial cells to secrete chemokines and antimicrobial peptides and promoting wound healing, but IL-17 also participates in autoimmunity and fibrosis (Conti and Gaffen, 2015; Majumder and McGeachy, 2021). IL-22 can promote the regeneration of epithelium and is involved in the host defense at barrier surfaces (Dudakov et al., 2015). The data indicate that adult-TD Th17 in the spleen may have a stronger Th17 function than neonatal-TD Th17. Though RORγt is a lineage-defining transcription factor that promotes the expression of IL-17 and IL-22 (Kumar et al., 2021), neonatal-TD Th17 expressed less *Il17a* and *Il22*, suggesting unknown factors that influence the expression of Th17 cytokines in these cells.

### Neonatal-TD Treg in the Peyer's patches also express higher levels of immunosuppressive genes

**Figure 3:**
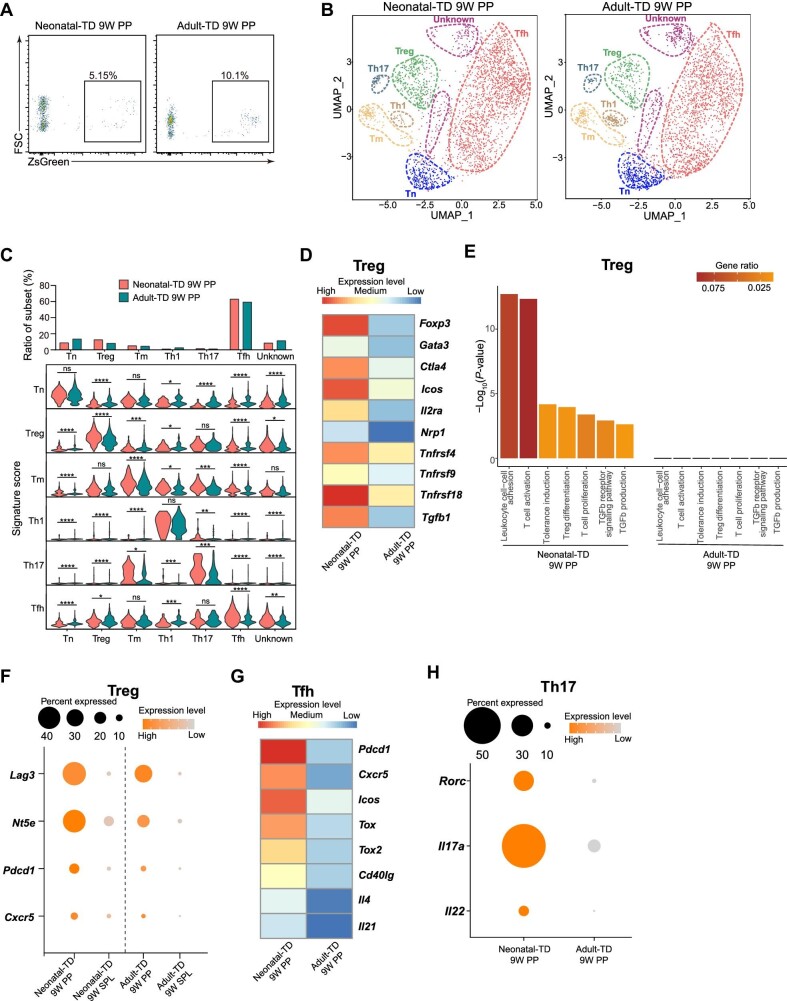
scRNA-seq reveals distinct subset distribution and gene expression patterns between neonatal-TD and adult-TD CD4^+^ T cells in the Peyer's patches after tracing for 9 weeks. (**A**) Flow cytometry analysis of 9-week-traced neonatal-TD and adult-TD ZsGreen^+^CD4^+^ T cells in the Peyer's patches. (**B**) UMAP visualization of scRNA-seq data from CD4^+^ T cells of 9-week-traced neonatal-TD (2912 cells) and adult-TD (11280 cells) in the Peyer's patches. Cells were segregated into seven clusters, including Tn, Treg, Tm, Th1, Th17, Tfh, and unknown clusters. The cell number of each sample was equalized. (**C**) The top bar graph indicates the frequency of cells in each cluster from the neonatal-TD and adult-TD groups, and the bottom stacked violin plot displays signature scores of each subset from different groups. (**D**) Heatmap reveals distinct expression levels of the signature genes (*Foxp3, Gata3, Ctla4, Icos, Il2ra, Nrp1, Tnfrsf4, Tnfrsf9, Tnfrsf18*, and *Tgfb1*) in Treg of neonatal-TD 9W PP and adult-TD 9W PP. (**E**) Selected GO terms in neonatal-TD 9W PP Treg compared with adult-TD 9W PP Treg. Bars represent the GO terms using a discrete color scale with gene ratio. Bar length represents –log_10_(*P*-value) of the GO terms. (**F**) Dot plot displays expression levels of the selected functional genes (*Lag3, Nt5e, Pdcd1*, and *Cxcr5*) in neonatal-TD and adult-TD Treg from the spleen and Peyer's patches. (**G**) Heatmap exhibits expression levels of the selected genes (*Pdcd1, Cxcr5, Icos, Tox, Tox2, Cd40lg, Il4*, and *Il21*) in Tfh of neonatal-TD 9W PP and adult-TD 9W PP. (**H**) Dot plot presents expression levels of the selected genes (*Rorc, Il17a*, and *Il22*) in Th17 of neonatal-TD 9W PP and adult-TD 9W PP.

We also traced CD4^+^ T cells from the neonatal-TD 9-week-traced Peyer's patches (neonatal-TD 9W PP, 3169 cells) and adult-TD 9-week-traced Peyer's patches (adult-TD 9W PP, 12000 cells) and analysed their single-cell transcriptomes (Supplementary Figure S3A). CD4^+^ZsGreen^+^ cells were sorted by FACS (Figure 3A). After filtering out the low-quality cells, cell doublets, and non-CD4^+^ T cells, the purity of CD4^+^ T cells (neonatal-TD 9W PP, 2912 cells; adult-TD 9W PP, 11280 cells) was examined by the expression of marker genes as previously described (Supplementary Figure S3B). The scRNA-seq data were further processed as previously described. The cluster distributions and sample origins were displayed in the UMAP space (Figure 3B). The clusters included Tn, Treg, Tm, Th1, Th17, Tfh, and unknown clusters. There was a clear shift of localization between neonatal-TD and adult-TD Tfh in the UMAP space. As previously mentioned, the signature genes of each subset were displayed by feature plots, which identified each T cell subset, and unknown clusters also expressed the same signature genes as in the spleen (Supplementary Figure S3B). Neonatal-TD cells showed an increase in the proportion of Treg (12.53% vs. 8.02%) and a decrease in the proportion of Tn (8.69% vs. 13.40%, Figure 3C). Treg, Th17, and Tfh signature scores were increased in neonatal-TD 9W PP cells, revealed by violin plot, suggesting the unique gene expression pattern in these three subsets of neonatal-TD cells (Figure 3C).

There was a significantly stronger expression of *Foxp3, Gata3, Ctla4, Icos, Il2ra, Nrp1, Tnfrsf4, Tnfrsf9, Tnfrsf18*, and *Tgfb1* in neonatal-TD Treg, similar to their counterparts in the spleen (Figure 3D). Several GO terms, including leukocyte cell–cell adhesion, T cell activation, tolerance induction, Treg differentiation, T cell proliferation, TGFβ signaling pathway, and TGFβ production, were enriched in neonatal-TD Treg, compared with their adult-TD counterparts (Figure 3E). TGFβ can promote Treg differentiation and suppress the activation and proliferation of effector T and B cells (Shevyrev and Tereshchenko, 2019; Sakaguchi et al., 2020). These data suggest that the 9-week-traced neonatal-TD Treg from both the spleen and Peyer,s patches have an enhanced immunosuppressive ability.

To analyse unique gene expression patterns in T cells from the Peyer's patches and spleen, we next performed data integration between scRNA-seq data of neonatal-TD and adult-TD T cells from the spleen and Peyer's patches (Supplementary Figure S3C). Compared with their spleen counterparts, *Lag3, Nt5e, Pdcd1*, and *Cxcr5* were highly expressed in both neonatal-TD and adult-TD Treg from the Peyer's patches (Figure 3F). Lag3 can bind to MHC-II on immature dendritic cells (DC) and suppress DC maturation (Liang et al., 2008). Lag3 can also inhibit CD4^+^ T cell activation through the recognition of peptide–MHC-II complexes (Maruhashi et al., 2018). CD73, which is encoded by *Nt5e*, can cause adenosine monophosphate to be degraded into extracellular adenosine. Increased adenosine concentrations can inhibit the activation and proliferation of T cells and suppress DC presentation of antigens (Linnemann et al., 2009; Ernst et al., 2010). We also found that neonatal-TD Treg in the Peyer's patches had higher expression levels of T follicular regulatory cell (Tfr) marker genes *Pdcd1* and *Cxcr5* (Ding et al., 2019). The interaction between the PD-1 of Treg and the PD-L1 of DC can generate tolerogenic DC (Gianchecchi and Fierabracci, 2018). These results imply the potential Tfr phenotype of neonatal-TD Treg from the Peyer's patches.

The expression levels of *Pdcd1, Cxcr5, Icos, Tox, Tox2, Cd40lg, Il4*, and *Il21*, which have important roles in Tfh maturation and function, were significantly increased in neonatal-TD Tfh (Figure 3G). PD-1 is essential for the positioning and function of Tfh, and the chemokine receptor Cxcr5 promotes B cell and T cell migration toward B cell follicles (Vinuesa et al., 2016; Shi et al., 2018). Costimulatory molecule ICOS promotes Tfh development and the maintenance of Tfh phenotype (Choi et al., 2011; Weber et al., 2015). Tox and Tox2 are transcription factors that can drive Tfh development by regulating chromatin accessibility (Xu et al., 2019). CD40L, IL-4, and IL-21 signals are required for the proliferation and differentiation of B cells (Crotty, 2019). These results reveal the potential stronger Tfh function of neonatal-TD cells.

The expression levels of *Il17a, Il22*, and *Rorc* were increased in neonatal-TD Th17 (Figure 3H). RORγt can promote the expression of IL-17 and IL-22 (Kumar et al., 2021). The increased expression levels of functional genes in neonatal-TD cells under healthy conditions may suggest the role of neonatal-TD Th17 in the protection of gut-associated lymphoid tissue.

### Neonatal-TD Treg from the spleen are more immunosuppressive than adult-TD Treg

**Figure 4:**
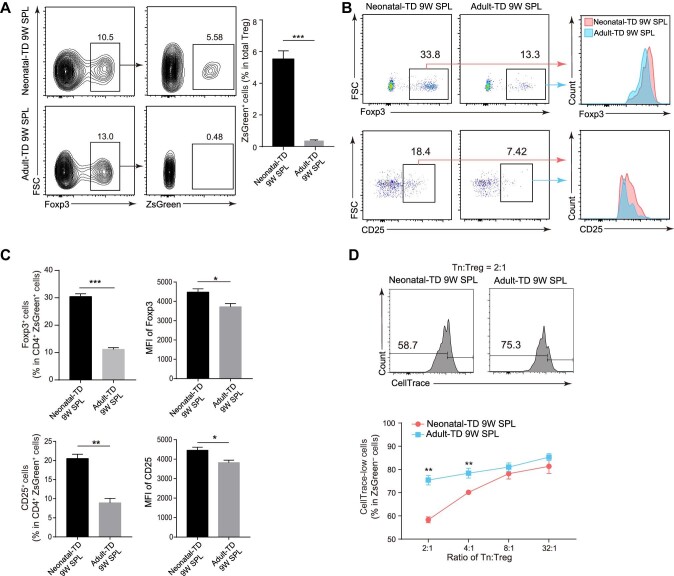
Neonatal-TD Treg exhibit a stronger immunosuppressive function than adult-TD Treg. (**A**) Flow cytometry (left) and statistical analysis (right) reveal the proportion of ZsGreen^+^Foxp3^+^ neonatal-TD and adult-TD Treg in total splenic Treg. All cells were gated from total CD4^+^ T cells. Mice were treated with tamoxifen during neonatal 0–2 days or at 6 weeks and traced for 9 weeks. (**B**) Flow cytometry analysis and MFI of Foxp3 and CD25 expression in 9-week-traced neonatal-TD/adult-TD CD4^+^ T cells from the spleen. Neonatal and adult mice (*n* = 3 mice per group) were treated with tamoxifen at neonatal 0–2 days or at 6 weeks and traced for 9 weeks. All cells were gated from CD4^+^ cells. (**C**) Statistical analysis for the ratio of Foxp3- or CD25-positive cells (left) and the MFI of Foxp3 and CD25 expression (right) in **B**. (**D**) The *in vitro* suppression assay of neonatal-TD and adult-TD Treg in the spleen. Flow cytometry (top) and statistical analysis (bottom) reveal the proportion of CellTrace-low cells in ZsGreen-negative Tn after a 3-day co-culture. Mice (*n* = 3 mice per group) were treated with tamoxifen during neonatal 0–2 days or at 6 weeks and traced for 9 weeks. CD4^+^CD25^+^ZsGreen^+^ Treg were sorted from the spleen of 9-week-traced neonatal-TD/adult-TD groups. CD4^+^ Tn were isolated from wild-type mice by magnetic beads and marked by CellTrace. Treg and Tn were then mixed and co-cultured in a round-bottom 96-well plate with anti-CD3/CD28 beads for 3 days.

The ability of Treg to inhibit T cell activation represents the strength of Treg in regulating the immune response. We found that the proportion of neonatal-TD Treg in total Treg was significantly higher than that of adult-TD Treg after tracing for 9 weeks (Figure 4A). This phenotype suggests that neonatal-TD Treg are difficult to be replaced by Treg generated afterward, which indicates that neonatal-TD Treg are more longevous than adult-TD Treg.

To further investigate the functional difference between neonatal-TD and adult-TD Treg, we analysed the expression levels of key proteins in neonatal-TD and adult-TD Treg after tracing for 9 weeks by flow cytometry analysis. We found that neonatal-TD T cells had higher proportions of both Foxp3^+^ and CD25^+^ cells, as well as higher mean fluorescent intensity (MFI) values of Foxp3 and CD25, than neonatal-TD Treg (Figure 4B and C), suggesting that neonatal-TD Treg have a reinforced suppressive ability compared with adult-TD Treg. Next, we co-cultured Tn and Treg *in vitro*. Neonatal-TD and adult-TD T cells were traced for 9 weeks (three mice in each group), and CD4^+^CD25^+^ZsGreen^+^ Treg of neonatal-TD and adult-TD were sorted by FACS, respectively. Then, neonatal-TD and adult-TD Treg were gradient-diluted from 1 × 10^4^ cells/well (Tn:Treg = 2:1) to 6.25 × 10^2^ cells/well (Tn:Treg = 32:1) and mixed with CellTrace-labelled Tn sorted from wild-type mice. These co-cultures were stimulated with anti-CD3/CD28 antibodies for 3 days and analysed by flow cytometry analysis. We found that mixing Tn with a high concentration of neonatal-TD Treg (Tn:Treg = 2:1) resulted in a lower proportion of CellTrace-low cells (58.7% vs. 75.3% in ZsGreen-negative cells), and the proportion increased as the Treg concentration decreased (Figure 4D). Based on these results, we demonstrate that neonatal-TD Treg in the mature immune system had a stronger T cell suppressive ability than adult-TD Treg.

### Newly egressed neonatal-TD and adult-TD T cells exhibit similar subset distribution and gene expression patterns

**Figure 5:**
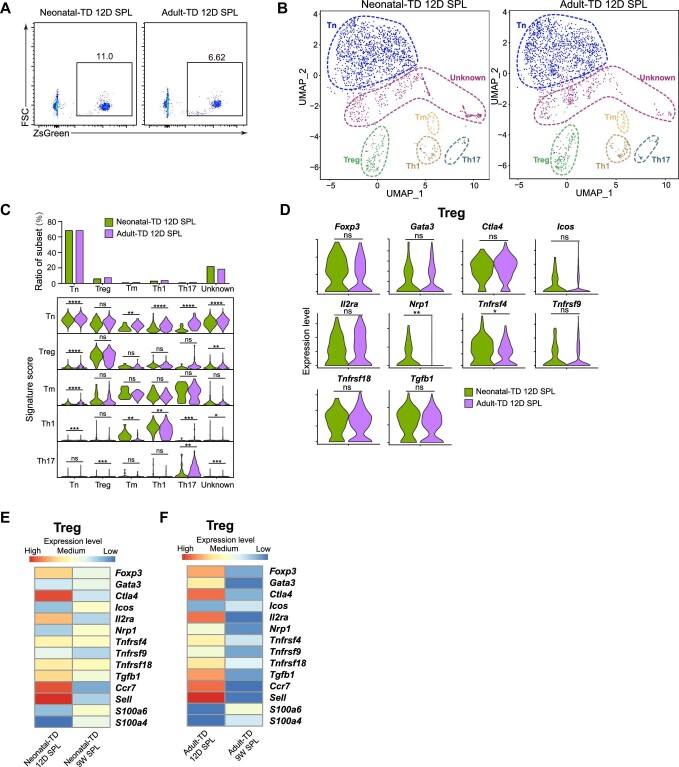
Twelve-day-traced neonatal-TD and adult-TD splenic T cells share similar subset distribution and gene expression patterns. (**A**) Flow cytometry analysis of 12-day-traced neonatal-TD and adult-TD ZsGreen^+^CD4^+^ T cells in the spleen. (**B**) UMAP visualization of scRNA-seq data from CD4^+^ T cells of 12-day-traced neonatal-TD SPL (1784 cells) and adult-TD SPL (4452 cells) groups. Cells were segregated into six clusters, including Tn, Treg, Tm, Th1, Th17, and unknown clusters. The cell number of each sample was equalized. (**C**) The top bar graph indicates the frequency of cells in each cluster from the neonatal-TD and adult-TD groups, and the bottom stacked violin plot displays signature scores of each subset from different groups. (**D**) Violin plots display distinct expression levels of the signature genes (*Foxp3, Gata3, Ctla4, Icos, Il2ra, Nrp1, Tnfrsf4, Tnfrsf9, Tnfrsf18*, and *Tgfb1*) in 12-day-traced neonatal-TD and adult-TD Treg. (**E**) Heatmap reveals distinct expression levels of the signature genes (*Foxp3, Gata3, Ctla4, Icos, Il2ra, Nrp1, Tnfrsf4, Tnfrsf9, Tnfrsf18, Tgfb1, Ccr7, Sell, S100a6*, and *S100a4*)between neonatal-TD 12D SPL Treg and neonatal-TD 9W SPL Treg. (**F**) Heatmap reveals distinct expression levels of the signature genes (*Foxp3, Gata3, Ctla4, Icos, Il2ra, Nrp1, Tnfrsf4, Tnfrsf9, Tnfrsf18, Tgfb1, Ccr7, Sell, S100a6*, and *S100a4*) between adult-TD 12D SPL Treg and adult-TD 9W SPL Treg.

To test whether the formation of features in neonatal-TD T cells is environment-dependent or cell-intrinsic, we compared the thymic emigrants from neonatal and adult mice. To be specific, neonatal-TD 12-day-traced (neonatal-TD 12D SPL, 1886 cells) and adult-TD 12-day-traced (adult-TD 12D SPL, 4655 cells) CD4^+^ T cells of the spleen, which were mildly affected by the environment, were analysed by scRNA-seq (Supplementary Figure S4A). CD4^+^ZsGreen^+^ cells were sorted by FACS (Figure 5A). After filtering out the low-quality cells, cell doublets, and non-CD4^+^ T cells, the purity of neonatal-TD (1784 cells) and adult-TD (4452 cells) cells was examined by the expression of marker genes as previously described (Supplementary Figure S4B). The scRNA-seq data were further processed as previously described. The cluster distributions and sample origins of CD4^+^ T cells from 12-day-traced neonatal-TD and adult-TD spleen were displayed in the UMAP space (Figure 5B). Six clusters were identified, including Tn, Treg, Tm, Th1, Th17, and unknown clusters. There was no obvious distinction in cell distribution between neonatal-TD and adult-TD cells. Interestingly, unknown clusters in the 12-day-traced groups not only expressed interferon-stimulated genes, cell cycle genes, and *Nme1*/*Mif*, but also contained cells highly expressing Myb or Egr1/2, which were not found in cells after long-term tracing (Supplementary Figure S4B). The signature genes of each subset were used to identify distinct T cell subsets, which revealed that neonatal-TD cells had similar proportions of Treg (5.81% vs. 7.39%), Tn, Tm, Th1, and Th17 to their adult counterparts (Figure 5C).

### Function and characteristics of neonatal-TD Treg are influenced by environmental factors

Unlike after the long-term tracing, expression levels of *Foxp3, Gata3, Ctla4, Icos, Il2ra, Tgfb1, Tnfrsf9*, and *Tnfrsf18* did not significantly differ between Treg in neonatal-TD and adult-TD 12-day-traced spleen, but the levels of *Tnfrsf4* and *Nrp1* were significantly increased in neonatal-TD 12D SPL Treg compared with adult-TD 12D SPL Treg (Figure 5D).

To distinguish subset distribution and gene expression patterns between neonatal-TD and adult-TD T cells after short- and long-term tracing, we performed data integration with scRNA-seq data of 12-day-traced/9-week-traced neonatal-TD and adult-TD CD4^+^ T cells from the spleen, and the cluster distributions and sample origins of T cells were displayed in the UMAP space (Supplementary Figure S4C). The data revealed that Treg of both neonatal-TD and adult-TD 12D SPL were similar to resting Treg based on their significantly increased *Sell* and *Ccr7* expression, while Treg of both neonatal-TD and adult-TD 9W SPL were more similar to activated Treg based on the increased expression of *S100a6* and *S100a4* (Figure 5E and F; Zemmour et al., 2018; Shevyrev and Tereshchenko, 2019). The expression levels of *Foxp3, Ctla4, Il2ra, Tnfrsf9*, and *Tgfb1* were significantly increased in Treg of both neonatal-TD and adult-TD 12D SPL, compared with their 9-week-traced counterparts. The expression levels of *Gata3, Tnfrsf4*, and *Tnfrsf18* were also significantly decreased in adult-TD 9W SPL Treg, compared with adult-TD 12D SPL Treg, but not significantly different between Treg of neonatal-TD 9W SPL and neonatal-TD 12D SPL. The expression levels of *Icos* and *Nrp1* were significantly increased in neonatal-TD 9W SPL Treg compared with neonatal-TD 12D SPL Treg, but not significantly different between Treg of adult-TD 9W SPL and adult-TD 12D SPL (Figure 5E and F). These results suggested that the expression levels of most Treg signature genes were decreased in 9-week-traced Treg compared with that in 12-day-traced Treg, both in neonatal-TD and adult-TD groups. However, neonatal-TD 9W SPL Treg showed a weaker decline in the expression of these genes, compared with adult-TD 9W SPL Treg. Our data reveal that the formation of unique features in neonatal-TD Treg after long-term tracing is possibly shaped by environmental effects.

## Discussion

Researchers have long been interested in the distinction between neonatal-TD and adult-TD T cells (Adkins et al., 2004; Basha et al., 2014). However, the subset distribution and gene expression features of neonatal-TD and adult-TD T cells still have not been revealed. By using the TOTT mouse model, we traced the T cells originating from the neonatal and adult periods and generated single-cell transcriptomes of the CD4^+^ T cells in the spleen and Peyer's patches after tracing for 9 weeks. Our results showed the enhanced expression of immunosuppressive molecules and function of 9-week-traced neonatal-TD Treg, demonstrating the more important role of neonatal-TD Treg in immunosuppression by flow cytometry analysis and *in vitro* co-culture. To reveal the environmental influence on the formation of neonatal-TD cell features, we analysed the single-cell transcriptomes of neonatal-TD and adult-TD CD4^+^ T cells in the spleen after short-term tracing. The 9-week-traced neonatal-TD T cells of both the spleen and Peyer's patches had an increased proportion of Treg and higer expression levels of Treg signature genes (*Foxp3, Gata3, Ctla4, Il2ra, Icos, Tgfb1, Tnfrsf9*, and *Tnfrsf18*), but 12-day-traced neonatal-TD T cells had the similar proportions of Treg and other subsets and expression levels of Treg signature genes, compared to their adult-derived counterparts. Our data suggest that environmental factors possibly play a key role in the formation of enhanced immunosuppressive ability of 9-week-traced neonatal-TD Treg.

The neonatal immune system performs a bias to immune tolerance (West, 2016; Yang et al., 2020). Treg are key regulators in the maintenance of immune tolerance and homeostasis (Lu et al., 2017). Treg produced during the neonatal age window are crucial for the establishment and maintenance of immune homeostasis during life (Yang et al., 2020). Previous evidence suggests the essential role of neonatal-TD Treg in promoting tolerance to antigens of commensal bacteria in mice (Scharschmidt et al., 2015, 2017). Similarly, thymus-derived Treg have an important role in the formation of self-tolerance in the liver (Li et al., 2020). Our data revealed the increased proportion and immunosuppressive ability of neonatal-TD Treg after long-term tracing, which suggested the enhanced function to control the immune response to self and foreign targets and prevent excessive or unnecessary immune activation. The mechanism of these features is still unknown, but further research in this area can improve the research of therapies for allergy and autoimmune diseases.

Our data revealed that most Treg signature genes were equally expressed in 12-day-traced neonatal-TD and adult-TD Treg, and only several genes were more expressed in 12-day-traced neonatal-TD Treg. But 9-week-traced neonatal-TD Treg had a stronger signature gene expression, compared with their adult counterparts. Treg signature gene expression levels were strongly reduced in adult-TD 12D SPL Treg compared to adult-TD 9W SPL Treg, but only mildly reduced in neonatal-TD 12D SPL Treg compared to neonatal-TD 9W SPL Treg. Our data revealed that *Nrp1* was highly expressed in both 12-day-traced and 9-week-traced neonatal-TD Treg. Several studies suggested that Treg can lose the expression of Foxp3 and exhibit an effector phenotype (Overacre and Vignali, 2016). Nrp1 plays a key role in the maintenance of the core program and immunosuppressive function of Treg (Delgoffe et al., 2013; Chuckran et al., 2020). Nrp1 is important for the stability of Treg by the SEMA4A-ligation of NRP1 (Delgoffe et al., 2013; Overacre-Delgoffe et al., 2017). *Nrp1*^–/–^ Treg in tumors adopt characteristic T helper lineage markers, which indicates that Nrp1 is also essential for Treg in tumors to maintain their phenotype and function (Delgoffe et al., 2013; Overacre-Delgoffe et al., 2017). One potential explanation for this phenotype is that the neonatal cell-intrinsic gene expression features in neonatal-TD cells, like *Nrp1*, may affect the gene expression pattern of neonatal-TD Treg during long-term environmental exposure. Neonatal Tn preferentially become Treg under the stimulation of TCR (Wang et al., 2010). Neonatal-TD and adult-TD Treg after long-term tracing also contained Treg differentiated from neonatal-TD and adult-TD Tn. Thus, another potential explanation is that the feature of neonatal-TD Treg is formatted under distinct environmental influences between the neonatal and adult periods, and the Treg derived from Tn may contribute to this process. In further research, we believe that transplanting the neonatal or adult thymus of the TOTT mouse model in the subcapsular space of a kidney of neonatal or adult mice is essential to reveal the environmental influence on the phenotype of neonatal-TD T cells. After transplantation, mice are treated with tamoxifen to trace the T cells produced from the transplanted thymus during this period. After 9 weeks, the ZsGreen^+^CD4^+^ T cells can be isolated and analysed by scRNA-seq and flow cytometry. By scRNA-seq, flow cytometry, and other technologies, we can directly test the environmental influence on the formation of neonatal-TD T cells.

Previous studies have found that neonatal T cell immunity was poised to Th2 and Treg responses, rather than Th1, Th17, and Tfh responses (Debock and Flamand, 2014). However, these studies could not reveal the phenotype of neonatal T cell differentiation *in vivo*, because of the lack of tools to distinguish the neonatal-TD T cells from other T cells. Our results revealed that neonatal-TD Tfh in the Peyer's patches have a gene expression pattern associated with maturer and stronger germinal center-B (GC-B) cell supporting the ability of Tfh. The neonatal immune system is initially contacted with the microbiota in the mucosa, and sIgA is one of the important weapons to establish a defense against these threats (Reboldi and Cyster, 2016; Renz et al., 2018). Improved maturation and GC-B cell-supporting ability can help with sIgA secretion and the establishment of gut immune homeostasis (Vinuesa et al., 2016). Neonatal-TD Th17 in the Peyer's patches also had higher *Il17a, Il22*, and *Rorc* expression levels than their adult-TD counterparts, which indicated enhanced Th17 function and better defensive ability against gut microbes (Kumar et al., 2021; Majumder and McGeachy, 2021). Neonatal-TD Th17 in the spleen had lower expression levels of *Il17a* and *Il22* but higher expression level of *Rorc* than adult-TD Th17, which may be due to the milder stimulation than in The Peyer's patches. Neonatal-TD Th1 in the spleen also exhibited reduced *Ifng* expression, which was consistent with previous studies on neonatal T cell immunity, but the enhanced gene expression associated with moving to inflamed tissues hinted at the possible distinction between the neonatal-TD and adult-TD Th1 responses (Forsthuber et al., 1996; Groom and Luster, 2011).

There were also some unclear subsets that highly expressed interferon-stimulated genes (*Ifit1* and *Isg15*), cell cycle genes (*Birc5* and *Mki67*), *Nme1* and *Mif, Egr1/2*, or *Myb*. The expression of interferon-stimulated genes indicated that these T cells have faced the challenge of IFN and are associated with the antiviral function (Schoggins, 2014). The expression levels of Nme1 and Mif are increased in activated T cells, and the results suggested that these T cells were stimulated by TCR (Bacher et al., 1996; Szabo et al., 2019). Myb plays a key role in the differentiation of T cells, and these T cells may represent a stage during T cell differentiation (Lieu et al., 2004). The transcription factors Egr1 and Egr2 are positive regulators of T cell activation and proliferation (Bettini et al., 2002; Du et al., 2014). *Egr1/2* or *Myb* was highly expressed in 12-day-traced T cells, but was not found in 9-week-traced cells. The phenotype may be caused by the milder environmental stimulation in 12-day-traced T cells.

This is the first study using scRNA-seq to compare the distinct cell fates between neonatal-TD and adult-TD CD4^+^ T cells bearing short-term or long-term environmental influences. Our work provides a panorama of neonatal-TD and adult-TD CD4^+^ T cells during short-term or long-term tracing, which can contribute to the research in the neonatal immunology field.

## Materials and methods

### Mice

*TCR*δ*^CreER^* allele was kindly gifted from Dr Yuan Zhuang's lab (Zhang et al., 2015). *R26^ZsGreen^* allele was purchased from the Jackson Laboratory (007906). Wild-type mice were purchased from Shanghai Lingchang Biotechnology Co. Ltd. Animals were bred and maintained under specific pathogen-free conditions in individually ventilated cages with a regular chow diet. All animal experiments were performed according to the guidelines for the use and care of laboratory animals as provided by Shanghai Jiao Tong University School of Medicine Institutional Animal Care and Use Committees.

### Tamoxifen treatment

Tamoxifen (Sigma-Aldrich, T5648-5G) was dissolved in corn oil (Aladdin, C116025) with a final concentration of 10 mg/ml. Neonatal-born mice were fed tamoxifen at 5 μl/day/mouse during 0–2 days. Tamoxifen treatment on adult mice (6 weeks) was induced by intraperitoneal injection, 200 μl/day/mouse for 3 days. Mice were sacrificed 12 days or 9 weeks after tamoxifen treatment. The tamoxifen-treated mice were abbreviated as 12-day-traced neonatal-TD/adult-TD or 9-week-traced neonatal-TD/adult-TD, respectively.

### Flow cytometry analysis and FACS

The thymus, spleen, and Peyer's patches were digested, washed with phosphate-buffered saline (PBS) containing 2% fetal bovine serum (FBS), and then filtered through a 70-μm cell strainer to prepare the single-cell suspension. For LPL, the intestine was dissected, and the remaining fat tissues and Peyer's patches were removed. Intestines were open longitudinally, cut into 2 cm pieces, and washed with PBS. Then, intestines were incubated in PBS containing 1 mM DTT, 30 mM EDTA, and 10 mM HEPES on a 200-rpm shaker at 37°C for 10 min, followed by incubation in PBS containing 30 mM EDTA and 10 mM HEPES at 37°C for 10 min. The intestine was then digested in RPMI1640 medium (Corning) containing Type VIII Collagenase (250 μg/ml; Sigma) and DNase I (90 μg/ml; Sigma) at 37°C in 5% CO_2_ incubator for 50 min. The digested tissues were homogenized by vigorous shaking and then filtered through a 70-μm cell strainer followed by further enrichment with 80% and 40% Percoll gradient.

Single-cell suspension was treated by Fc-Block (Biolegend, 156603) for 15 min at 4°C. For surface protein staining, cells were incubated with an antibody cocktail for 30 min at 4°C. To stain for transcription factors, cells following surface protein staining were treated with Foxp3/Transcription Factor Staining Buffer Set (Thermo Fisher, 00-5523-00) for 60 min at 4°C. Then, the cell suspensions were analysed according to a standard protocol by BD Fortessa X20 (BD Biosciences). The antibodies used in this study were as follows: anti-TCRγδ (Thermo Fisher, 12-5711-81), anti-TCRβ (Biolegend, 100438), anti-CD4 (Biolegend, 100438), anti-CD8 (Biolegend, 100752), anti-CD25 (Thermo Fisher Scientific, 17-0251-82), anti-Foxp3 (Thermo Fisher Scientific, 12-4771-82), and anti-CD44 (BD Biosciences, 560570). LIVE/DEAD™ Fixable Near-IR Dead Cell Stain Kit was used to detect dead cells (Thermo Fisher Scientific, L34976). ZsGreen expression was detected by FITC channel.

Neonatal tracing and adult tracing mice (up to five in each group for scRNA-seq) were sacrificed and single-cell suspensions were prepared as previously described. Single-cell suspensions from each group were mixed and stained, and then CD4^+^ZsGreen^+^ T cells were sorted by BD FACS Aria III (BD Biosciences) based on the expression of CD4 and ZsGreen. Because flow cytometry results revealed that all CD4^+^ZsGreen^+^ cells were TCRβ^+^ and TCRγδ^−^, we believed that γδT cells were removed from our scRNA-seq data.

### scRNA-seq and data analysis

scRNA-seq datasets were produced using Single Cell 3′ Chip (9-week-traced) or Single Cell 5′ Chip (12-day-traced) (10x Genomics, 1000009, 1000075, or 1000298). cDNA molecules were pre-amplified, fragmented, end-repaired, and ligated with adaptors as per the manufacturer's protocol in a single Illumina HiSeq X ten or Novaseq 6000 cartridge. All libraries were quantified by Qubit and the size profiles of the pre-amplified cDNA and sequencing libraries were examined using an Agilent 2100 BioAnalyzer. Raw sequencing data were first de-multiplexed using Illumina bcl2fastq software to generate separate paired-end read files. Then, Cell Ranger software (10x Genomics) was used to perform alignment and transcript quantification.

R package Seurat (version 3.2.3; Butler et al., 2018) was used to explore QC metrics and filter cells: 200 < gene counts per cell < 2500 and the percentage of mitochondrial genes < 8%. B cells and CD8^+^ cells were removed by the expression of *Cd79a*/*Cd79b* and *Cd8a*/*Cd8b*. The data were normalized with the ‘NormalizeData’ function (normalization.method = ‘LogNormalize’, scale.factor = 10000). After gene expression normalization and PCA, filtered count matrices were combined with Harmony package (version 1.0; Korsunsky et al., 2019). Dimension reduction was then performed using Harmony-corrected principal components. ‘FindNeighbors’ function was used to get the nearest neighbors for graph clustering based on principal components, and ‘FindCluster’ was used to obtain cell subtypes. Then, cells were visualized with the UMAP algorithm. The first 40 principal components were used to generate an initial clustering using the Seurat community detection algorithm to identify cell clusters. The ‘FindAllmarkers’ function was used to identify genes distinctly expressed between samples or subsets. Following parameters were used to calculate variable genes: min.pct = 0.02, return.thresh = 0.01, logfc.threshold = 0.2. *P*-values of genes distinctly expressed between samples or subsets were determined by a non-parametric Wilcoxon rank-sum test, adjusted *P*-values (*Padj*) were based on Bonferroni correction. *Padj* < 0.05 was considered significant (*Padj < 0.05; **Padj < 0.01; ***Padj < 0.001, ****Padj < 0.001). Signature scores were calculated with ‘AddModuleScore’ in Seurat, for each T cell subset. *P*-values of signature scores were also calculated by a non-parametric Wilcoxon rank-sum test. The control features were selected from the feature genes of each subset. Feature plots, violin plots, dot plots, and heatmaps were also generated using ‘Seurat’ implemented functions. All gene expressions with significant differences were tested by a non-parametric Wilcoxon rank-sum test (*P* < 0.05).

The GO analysis was done by R package ClusterProfiler (version 3.14.3; Yu et al., 2012). Following parameters were used to calculate GO terms: ont = ‘ALL’, pAdjustMethod = ‘BH’, pvalueCutoff = 0.05, qvalueCutoff = 0.2. Statistical analysis was performed by hypergeometric distribution.

### In vitro Treg assay

Referring to the previous protocol (Collison and Vignali, 2011), after tracing for 9 weeks, neonatal or adult tamoxifen-treated mice (3 mice per group) were sacrificed, and CD4^+^ T cells from the spleen were mixed and then enriched by MACS (STEMCELL, #19852). Subsequently, CD4^+^CD25^+^ZsGreen^+^ cells were sorted by flow cytometry from enriched CD4^+^ T cells based on the expression of CD4, CD25, and ZsGreen. Meanwhile, naïve CD4^+^ T cells were enriched from wild-type mice by MACS (STEMCELL, #19765) and then marked by CellTrace Blue (2.5 μg/ml in PBS, 20 min in 37°C, Thermo Fisher Scientific, C34568). Treg were gradient-diluted into 96-well plates from 1 × 10^4^ cells/well, and Tn were titrated into plates by 2 × 10^4^ cells/well. The final culture system was 0.5 μl/well Dynabeads™ Mouse T-Activator CD3/CD28 (Thermo Fisher Scientific, 11456D) in T cell medium (RPMI1640, Penicillin/Streptomycin, 10% FBS). Cells were cultured at 37°C for 3 days before flow cytometry assay.

### Statistical analysis

Statistical analysis was performed using GraphPad Prism 7.0 software. Data are represented as mean ± standard error of the mean. The data were compared using a two-tailed unpaired Student's *t*-test. *P* < 0.05 was considered significant (*P < 0.05; **P < 0.01; ***P < 0.001).

### Data and code availability

The data that support the findings of this study have been deposited into CNGB Sequence Archive (CNSA) of China National GeneBank DataBase (CNGBdb) with the accession number CNP0003502. Codes were implemented in R 3.6.3 and are deposited in https://github.com/yuhenghan/Neonatal-TD_Adult-TD/Codes/V.1.0.0.

## Supplementary Material

mjac072_Supplemental_Files
