# Supplementary material for: scRNA-seq profiling of neonatal and adult thymus-derived CD4+ T cells by a T cell origin-time tracing model
Source: J Mol Cell Biol. 2022 Dec 20;14(12):mjac072. doi: 10.1093/jmcb/mjac072 (PMC10117164; doi:10.1093/jmcb/mjac072)
Supplement: mjac072_Supplemental_Files [file mjac072_supplemental_files.zip › SI Figure 1209.pdf]

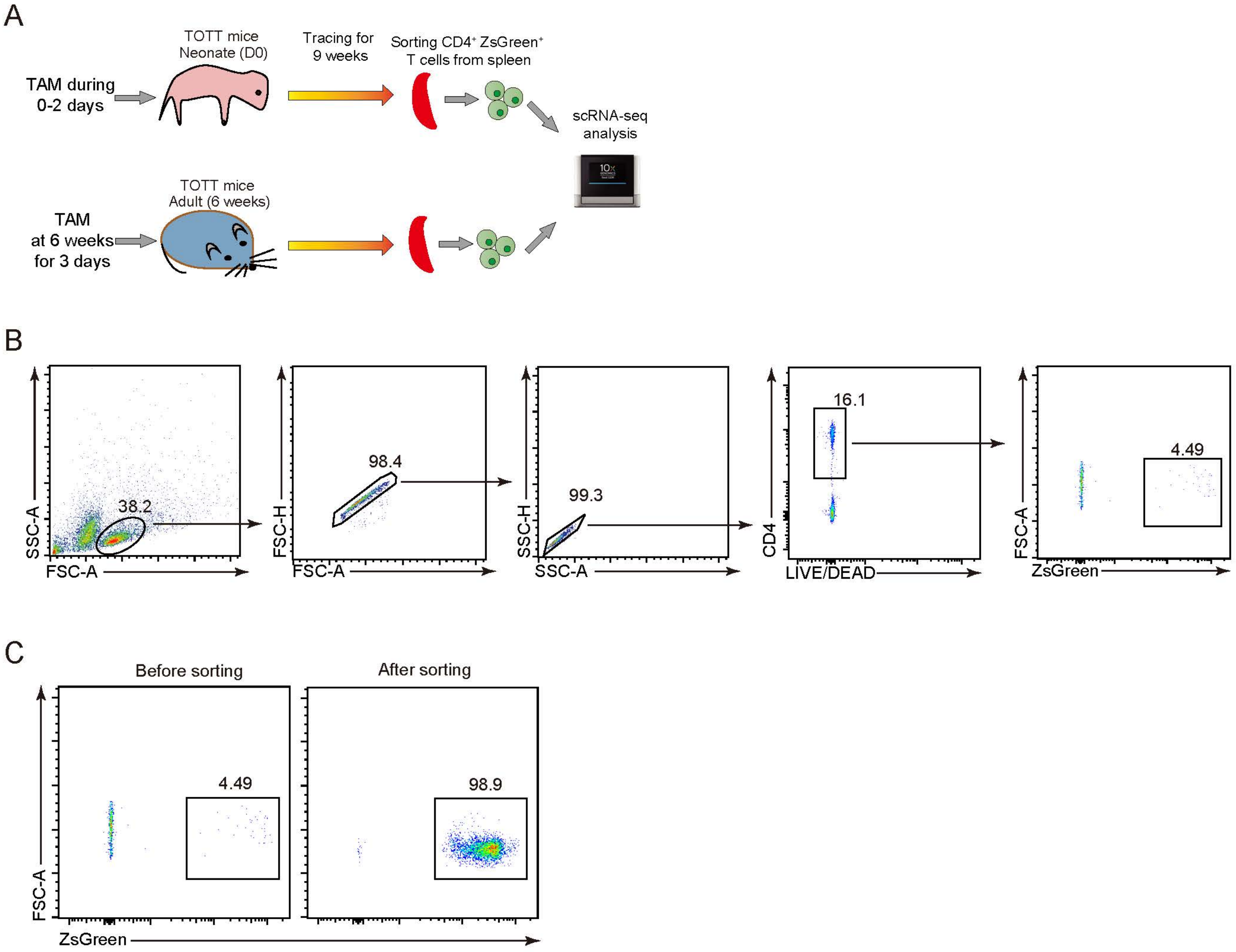

Supplementary Figure S1 Experimental design of FACS for scRNA-seq

(A). Workflow of the scRNA-seq analysis for neonatal thymus-derived (neonatal-TD) and adult-TD $CD4^+$ T cell in spleen after tracing for 9 weeks. T cell origin time tracing (TOTT) mice were treated with tamoxifen during neonatal 0-2 days or 6 weeks and traced for 9 weeks.

(B). Gating strategy for $CD4^+$ $ZsGreen^+$ T cells.

(C). Purities of $CD4^+$ $ZsGreen^+$ T cells before and after sorting were shown in the spleen of neonatal tracing 9 weeks group

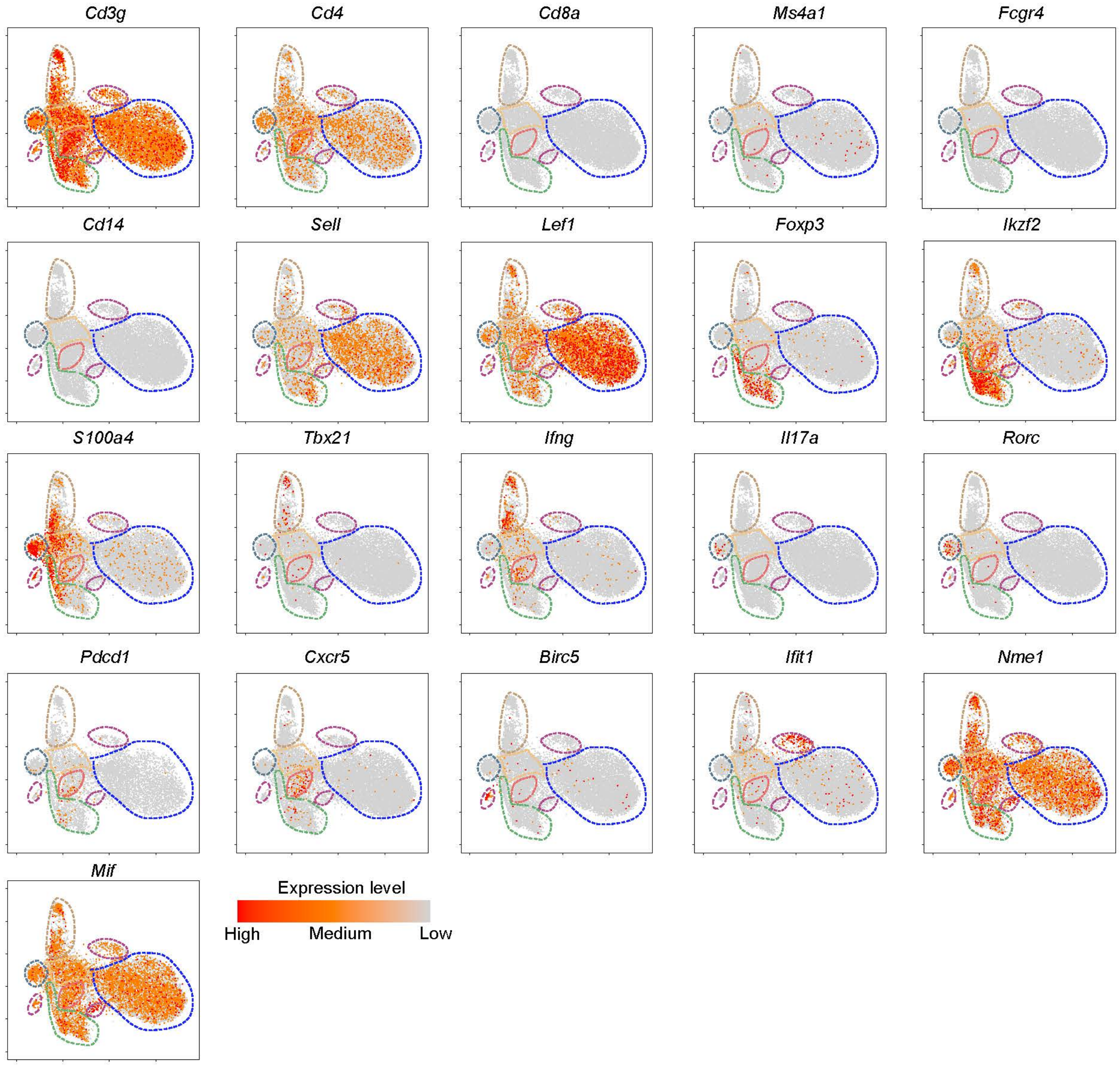

Supplementary Figure S2 Selected gene expression analysis of neonatal thymus-derived (neonatal-TD) and adult-TD CD4$^+$ T cells in spleen after tracing for 9 weeks, Related to Figure 2.

Feature plots of key genes (*Cd3g*, *Cd4*, *Cd8a*, *Ms4a1*, *Fcgr4*, *Cd14*, *Sell*, *Lef1*, *Foxp3*, *Ikzf2*, *S100a4*, *Tbx21*, *Ifng*, *Il17a*, *Rorc*, *Pdcd1*, *Cxcr5*, *Birc5*, *Ifit1*, *Nme1*, and *Mif*) expression in each subset of CD4$^+$ T cells from 9-week-traced neonatal-TD/adult-TD groups in spleen (SPL), as in Figure 2B.

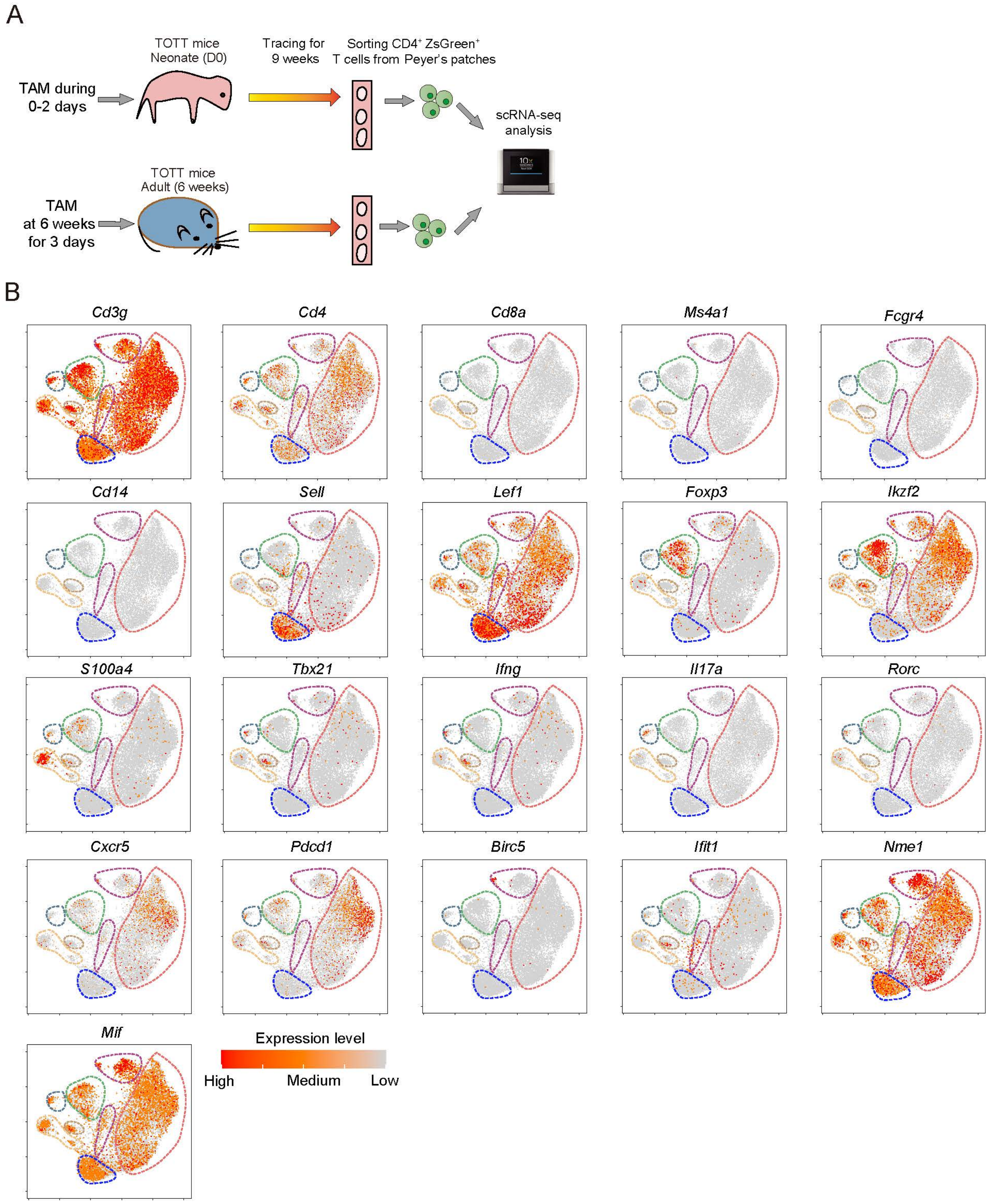
A
TOTT mice
Neonate (D0)
Tracing for
9 weeks
Sorting CD4+ ZsGreen+
T cells from Peyer's patches
TAM during
0-2 days
scRNA-seq
analysis
TOTT mice
Adult (6 weeks)
TAM
at 6 weeks
for 3 days
B
Cd3g
Cd4
Cd8a
Ms4a1
Fcgr4
Cd14
Sell
Lef1
Foxp3
Ikzf2
S100a4
Tbx21
Ifng
Il17a
Rorc
Cxcr5
Pdcd1
Birc5
Ifit1
Nme1
Mif
Expression level
High
Medium
Low

C

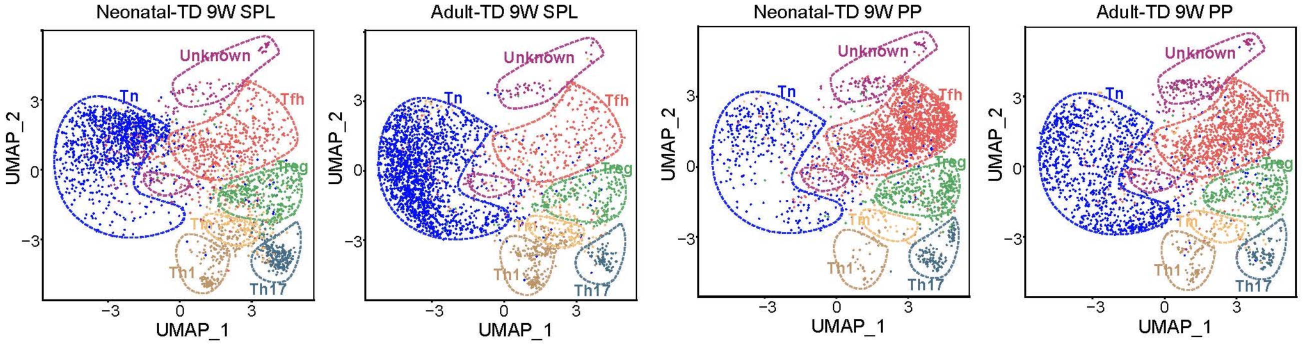

Supplementary Figure S3 Selected gene expression analysis of neonatal thymus-derived (neonatal-TD) and adult-TD $CD4^+$ T cells in Peyer's patches (PP) after tracing for 9 weeks, Related to Figure 3

(A). Workflow of the scRNA-seq analysis for neonatal-TD and adult-TD $CD4^+$ T cell in PP after tracing for 9 weeks. T cell origin time tracing (TOTT) mice were treated with tamoxifen during neonatal 0-2 days or 6 weeks and traced for 9 weeks.

(B). Feature plots of key genes (*Cd3g*, *Cd4*, *Cd8a*, *Ms4a1*, *Fcgr4*, *Cd14*, *Sell*, *Lef1*, *Foxp3*, *Ikzf2*, *S100a4*, *Tbx21*, *Ifng*, *Il17a*, *Rorc*, *Pdcd1*, *Cxcr5*, *Birc5*, *Ifit1*, *Nme1*, and *Mif*) expression in each subset of $CD4^+$ T cells from 9-week-traced neonatal-TD/adult-TD PP groups, as in Figure 3B.

(C). UMAP visualization of scRNA-seq data from $CD4^+$ T cells of 9-week-traced neonatal-TD spleen (SPL, 3561 cells)/adult-TD spleen SPL (10642 cells) and 9-week-traced neonatal-TD PP (2912 cells)/adult-TD PP (11280 cells). The cell number of each sample had been equalized.

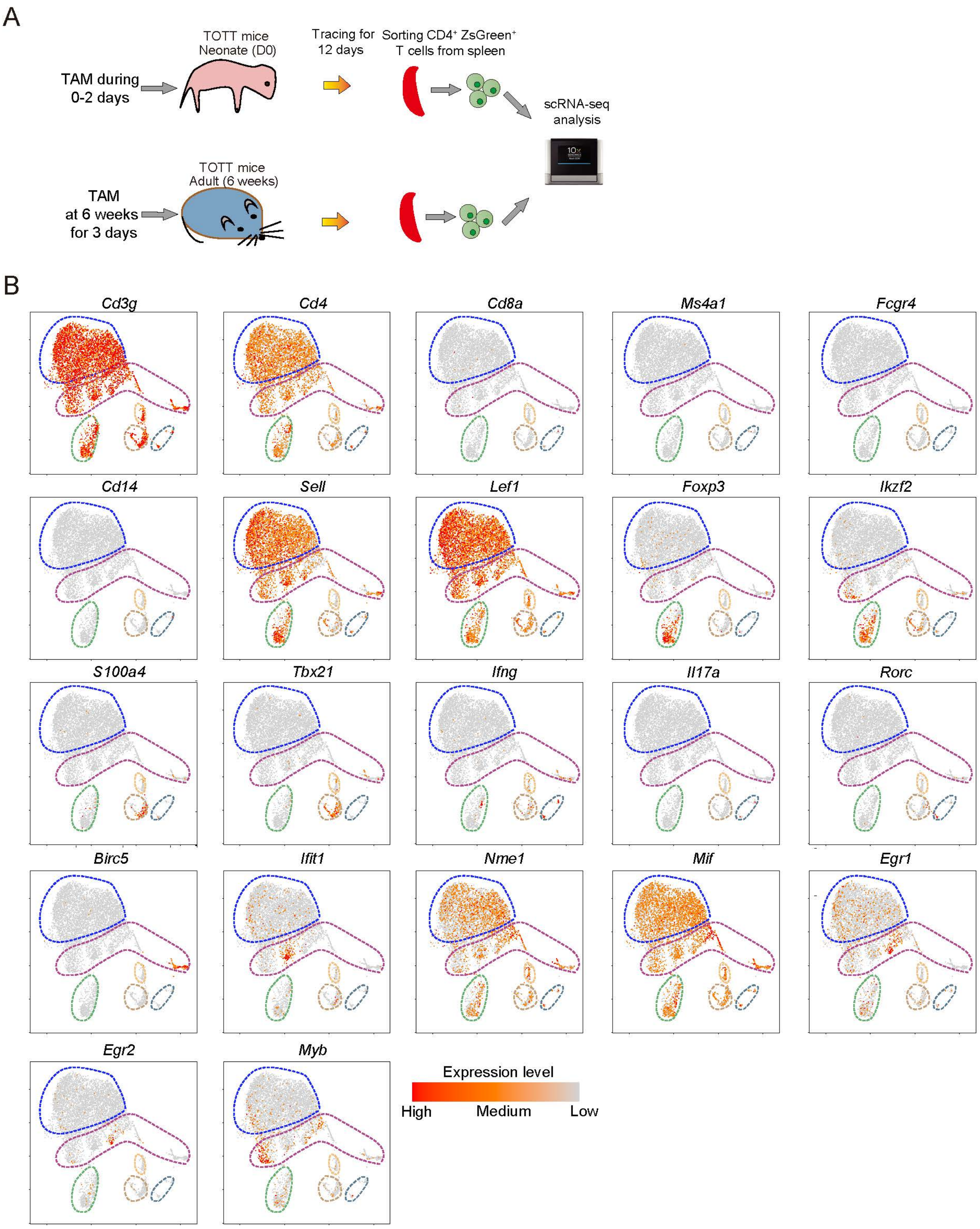
A
TOTT mice
Neonate (D0)
Tracing for
12 days
Sorting CD4+ ZsGreen+
T cells from spleen
TAM during
0-2 days
scRNA-seq
analysis
TOTT mice
Adult (6 weeks)
TAM
at 6 weeks
for 3 days
B
Cd3g
Cd4
Cd8a
Ms4a1
Fcgr4
Cd14
Sell
Lef1
Foxp3
Ikzf2
S100a4
Tbx21
Ifng
Il17a
Rorc
Birc5
Ifit1
Nme1
Mif
Egr1
Egr2
Myb
Expression level
High
Medium
Low

C

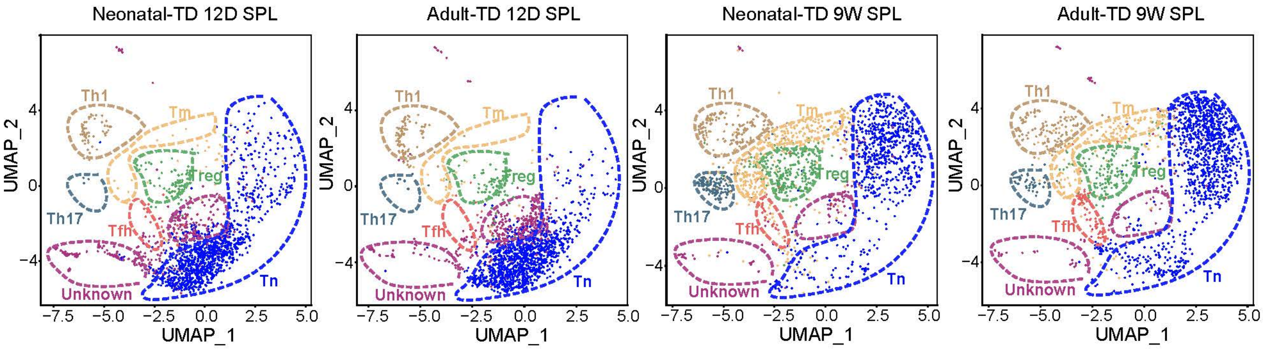

Supplementary Figure S4 Selected gene analysis of neonatal thymus-derived (neonatal-TD) and adult-TD CD4$^+$ T cells in the spleen after tracing for 12 days, Related to Figure 5

(A). Workflow of the scRNA-seq analysis for neonatal-TD and adult-TD CD4$^+$ T cell in spleen (SPL) after tracing for 12 days. T cell origin time tracing (TOTT) mice were treated with tamoxifen during neonatal 0-2 days or 6 weeks and traced for 12 days.

(B). Feature plots of key genes (*Cd3g*, *Cd4*, *Cd8a*, *Ms4a1*, *Fcgr4*, *Cd14*, *Sell*, *Lef1*, *Foxp3*, *Ikzf2*, *S100a4*, *Tbx21*, *Ifng*, *Il17a*, *Rorc*, *Birc5*, *Ifit1*, *Nme1*, *Mif*, *Egr1*, *Egr2*, and *Myb*) expression in each subset of CD4$^+$ T cells from 9-week-traced neonatal-TD/adult-TD spleen groups, as in Figure 5B.

(C). UMAP visualization of scRNA-seq data from CD4$^+$ T cells of 12-day-traced neonatal-TD SPL (1784 cells)/adult-TD SPL (4452 cells) and 9-week-traced neonatal-TD SPL (3561 cells)/adult-TD SPL (10642 cells). The cell number of each sample had been equalized.

Supplementary table 1 The list of genes used in signature score analysis

Tn	Treg	Tm	Th1	Th17	Tfh
Sell	*Foxp3*	*S100a4*	*Tbx21*	*Tmem176a*	*Pdcd1*
Dapl1	*Il2ra*	*S100a6*	*Ifng*	*Tmem176b*	*Cxcr5*
Ccr7	*Ikzf2*	*Cd44*	*Xcl1*	*Il17a*	*Tox2*
Lef1	*Ctla4*	*S100a11*	*Nkg7*	*Rorc*	*Tox*
Il7r	*Tnfrsf4*	*Itgb1*	*Ccl5*		*Tnfsf8*
	Tnfrsf18	*Ahnak*			*Icos*
					Cd40lg
